# Genetic Characterization of a Novel Iflavirus Associated with Vomiting Disease in the Chinese Oak Silkmoth *Antheraea pernyi*

**DOI:** 10.1371/journal.pone.0092107

**Published:** 2014-03-17

**Authors:** Peng Geng, Wenli Li, Lan Lin, Joachim R. de Miranda, Scott Emrich, Lijia An, Olle Terenius

**Affiliations:** 1 School of Life Science and Biotechnology, Dalian University of Technology, Dalian, Liaoning, People's Republic of China; 2 School of Life Science and Bio-pharmaceutics, Shenyang Pharmaceutical University, Shenyang, People's Republic of China; 3 Department of Ecology, Swedish University of Agricultural Sciences (SLU), Uppsala, Sweden; 4 Department of Computer Science and Engineering, University of Notre Dame, Notre Dame, Indiana, United States of America; University of South Florida College of Medicine, United States of America

## Abstract

Larvae of the Chinese oak silkmoth (*Antheraea pernyi*) are often affected by AVD (*A. pernyi* vomiting disease), whose causative agent has long been suspected to be a virus. In an unrelated project we discovered a novel positive sense single-stranded RNA virus that could reproduce AVD symptoms upon injection into healthy *A. pernyi* larvae. The genome of this virus is 10,163 nucleotides long, has a natural poly-A tail, and contains a single, large open reading frame flanked at the 5′ and 3′ ends by untranslated regions containing putative structural elements for replication and translation of the virus genome. The open reading frame is predicted to encode a 3036 amino acid polyprotein with four viral structural proteins (VP1-VP4) located in the N-terminal end and the non-structural proteins, including a helicase, RNA-dependent RNA polymerase and 3C-protease, located in the C-terminal end of the polyprotein. Putative 3C-protease and autolytic cleavage sites were identified for processing the polyprotein into functional units. The genome organization, amino acid sequence and phylogenetic analyses suggest that the virus is a novel species of the genus *Iflavirus*, with the proposed name of *Antheraea pernyi* Iflavirus (ApIV).

## Introduction

**Figure 1:**
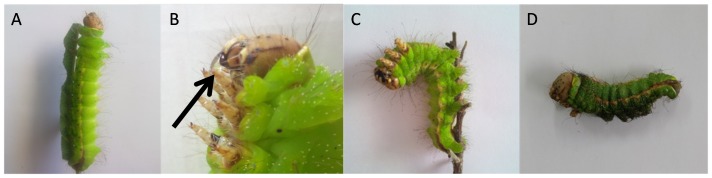
5^th^ instar larvae with progressing AVD symptoms. (A) Healthy larva, (B) vomiting larva with arrow pointing at vomit, (C) larva hanging from branch and (D) shortened body of dead larva.

*Antheraea pernyi* Vomit Disease (AVD) is a common disease of the Chinese oak silkmoth, *Antheraea pernyi* [1]. AVD is widely distributed in the cold mountainous regions in the north of China, such as the Liao Ning, Ji Lin, and the Hei Longjiang provinces, but also found in the much warmer He Nan province in the south. It occurs mainly in the 5^th^ instar, especially close to the cocooning period. Infected larvae will become sluggish and the color of their pygidia turns black. The most typical symptom of early AVD is a white liquid vomited up from the midgut. Infected larvae lose the ability to hold on to twigs and drop to the ground or remain dangling from the branches by their posterior end (Fig. 1). As the disease progresses, the larvae stop eating, resulting in shortened bodies that with the death of the larvae turn dark as decay sets in. Since this decay does not turn cankerous or fishy, as happens when the larvae are infected by bacteria, the causative agent of this disease was already in 1986 suspected to be a virus [1].

In an unrelated transcriptome analysis of *A. pernyi* larvae, a considerable number of cDNA sequences were obtained, assembling into a single contig of about 3 kb, with high similarity to the RNA-dependent RNA polymerase (RdRp) of the Iflaviruses. Using an RT-PCR assay based on this contig, a small set of AVD-affected individuals were found to contain Iflavirus RNA, which triggered the present investigation.

The Iflaviridae is a family of Group IV, positive-sense single-stranded RNA insect-infecting viruses within the order Picornavirales, containing a single genus *Iflavirus* [2]. *Iflavirus* particles contain a single-stranded RNA genome of positive polarity that encodes a single, large polyprotein, which is post-translationally processed into viral proteins essential for its replication, packaging and transmission [3]. The 5′UTR includes an internal ribosome entry site (IRES) structure needed for the cap-independent translation [4]–[6]. Downstream of the 5′ UTR is a single large open reading frame (ORF) that encodes both structural (5′terminus) and non-structural (3′ terminus) proteins. The ORF is followed by a 3′UTR, which is followed by a poly(A) tail [3].

Here, we report the nucleotide sequence, genomic organization and phylogenetic placement of this novel Iflavirus, its link to AVD, and its geographic and life-stage distribution in *A. pernyi*.

## Materials and Methods

### Sample origins

All the *A. pernyi* samples were collected with authorization from the Chinese Academy of Agricultural Sciences. The study was approved by the Ethics Committee of Dalian University of Technology.

**Figure 2:**
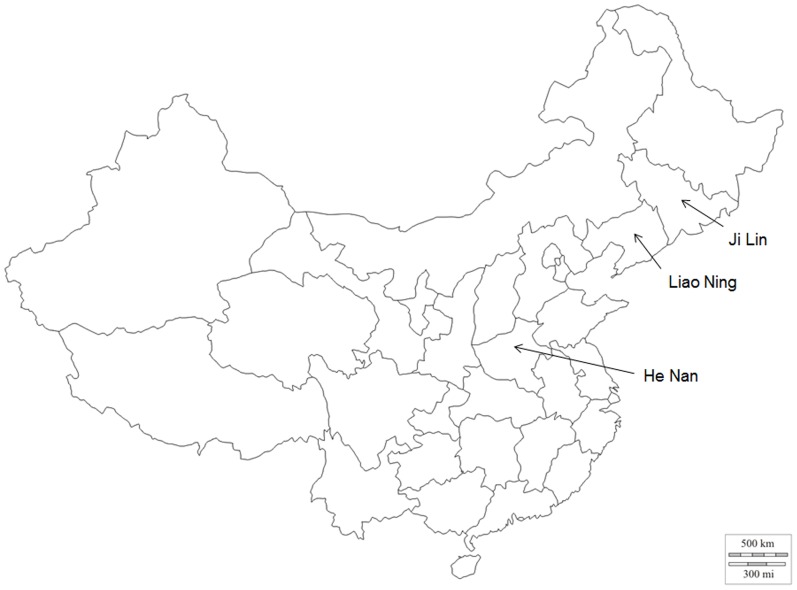
Sample locations for analysis of ApIV regional distribution. Original map from http://www.d-maps.com/carte.php?num_car=17501&lang=en.

The samples included in the study were collected in October 2012 from three provinces in the People's Republic of China (Liao Ning, Ji Lin and He Nan; Figure 2). He Nan is located in the middle of China. It has a distinct seasonal climate characterized by hot, humid summers and generally cool to cold, dry winters. Temperatures average around the freezing mark in January and 27 to 28 °C in July. A great majority of the annual rainfall occurs during the summer. There are 240 frost-free days annually. Liao Ning and Ji Lin are in the east of China. The annual average temperature in Liaoning is about 9 °C. January is the coldest month with the lowest temperature being −11 °C, while the highest temperature in July is 24 °C. Ji Lin has a northerly continental monsoon climate, with long, cold winters and short, warm summers. January mean temperature is −17.3 °C, and July mean temperature is 22.8 °C.

A total of 144 *A. pernyi* samples (80 eggs from 12 moths, 12 larvae, 40 chrysalises and 12 adult moths) were collected. The samples from the Liao Ning province were kept in a rearing chamber at 25±3°C with 70±5% relative humidity, with fresh Chinese oak leaves for feeding the larvae (adult moths have a life-span of a few days and do not eat). The samples from the Ji Lin and He Nan provinces were frozen after collection and stored at −80 °C until processing.

### Discovery, cloning and sequencing

The cDNA from an unrelated transcriptome project was examined by Illumina sequencing, generating 10,588 contigs whereof one 3 kb contig aligned to iflaviruses. This contig allowed us to design the initial primers for the cloning and sequencing of the RdRp region of the new iflavirus. The Iflavirus genome is naturally poly-adenylated, such that the Illumina sequences comprising the initial 3 kb contig were all located towards the 3′ end of the virus genome. The remainder of the genome was determined through primer-walking, using a series of forward and reverse primers (figure S1). The 5′ and 3′ ends were assessed by rapid amplification of cDNA ends (RACE) methodology using a FirstChoice RLM-RACE Kit (Invitrogen), and two sets of specific primers. All amplified fragments were purified, cloned into the pMD19-Tvector (Takara) and sequenced using Sanger sequencing technology.

### Sequence and phylogenetic analyses

The nucleotide and deduced amino acid sequences of the virus genome were scanned for functional domains and putative proteolytic processing sites. The secondary structure of the 5′ UTR was predicted using the MFOLD program [7] and rendered visually using the RnaViz 2.0 [8] and ViennaRNA [9] software packages.

**Figure 3:**
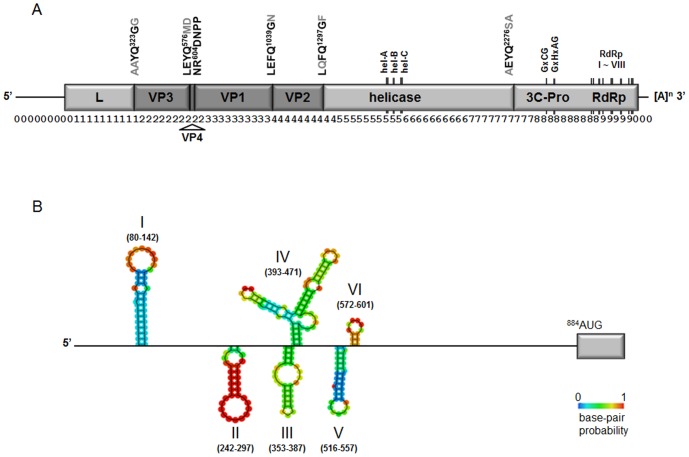
Schematic diagram of the genome organization of ApIV. A, Indicated are the structural proteins (VP1–VP4; dark grey) and the non-structural proteins (light grey); the putative protease cleavage sites (conserved amino acids in black font; variable amino acids in grey font); the conserved functional domains of the helicase, 3C-protease and RNA-dependent RNA polymerase (RdRp); the 5′UTR and 3′UTR and the poly-A tail. The labelling of the structural proteins follows Roberts and Groppelli [38]. The numbers below the genome map mark 100-nucleotide intervals. B, The predicted secondary structure of the ApIV 5′UTR. Indicated are six dominant stable secondary structures (I-VI) with the stability of each structure indicated by the heat map. The locations of the structures correspond to their nucleotide positions in the 5′UTR.

The amino acid sequence was aligned to homologous sequences from other Iflaviruses, as well as representative Dicistroviruses and Picornaviruses, using the DNAMAN program. Sections of this alignment surrounding the conserved domains of the helicase (Hel), 3C-protease (3C-Pro) and the RNA-dependent RNA polymerase (RdRp) were isolated for use in phylogenetic analysis, using Maximum Likelihood criteria as implemented by MEGA5 [10]. The final dataset comprised a total of 328 characters (including gaps), corresponding to amino acids 1615–1627 (Hel-A); 1647–1670 (Hel-B); 1700–1722 (Hel-C); 2457–2463 and 2471–2479 (3C-protease); 2698–2797 (RdRp-I, -II, -III and -IV); 2832–2870 (RdRp-V); 2881–2927 (RdRp-VI); 2934–2948 (RdRp-VII) and 2957–2975 (RdRp-VIII) of the new iflavirus genome (Figure 3A; Figure S2).

Initial tree(s) for the heuristic search were obtained automatically by applying Neighbor-Join and BioNJ algorithms to a matrix of pairwise distances estimated using a JTT model, and then selecting the topology with superior log likelihood value. The tree is drawn to scale, with branch lengths measured in the number of substitutions per site. Statistical support for the partitions was determined through bootstrap analyses involving 500 replicates. The full names and GenBank accession numbers of the viruses used in the phylogeny are found in Table S1. The 420 bp nucleotide sequences of the Helicase region and 2129 bp sequences of the capsid region from 9 isolates from the three provinces were aligned and scanned for variants, to obtain a measure of the natural geographic variability of the virus and the reliability of the diagnostic RT-PCR assays.

### ApIV propagation, purification and verification

Whole bodies of naturally AVD-affected larvae were dissected and homogenized by grinding in 2.5 ml PBS per 1 g of tissue). The homogenized extract was injected into healthy pupae (50 μl/pupae) which were incubated as described above. Three days post-injection, the hemolymph of infected pupae was collected and filtered using a fine-mesh nylon cloth to remove debris. The filtered extract was layered on top of a 25%:56% discontinuous sucrose gradient made in 1xPBS and centrifuged at 45,000 g for 2 hours at 4°C. The virus-containing fraction was collected at the middle interphase, between the 25% and 56% sucrose layers, using a needle. The presence of ApIV RNA in the purified fraction was confirmed by RT-PCR. The presence of Iflavirus-like particles in the purified fraction was determined by Transmission Electron Microscopy (TEM), using a JEM-1200EX transmission electron microscope.

Also, purified ApIV was injected into 5^th^ instar larvae. ApIV was diluted in sterile PBS and the dose was 5 μg/larva (which is estimated to about 4*10^11^ copies per larva). A mock-inject group was injected with sterile PBS.

### RNA extraction and cDNA synthesis

Geographic field isolates of AVD-symptomatic and asymptomatic larvae; eggs, pupae (chrysalis), adults and adult integument; as well as dissected chrysalis tissues, were homogenized by grinding the tissues in liquid nitrogen. Total RNA was extracted from 100 mg of each sample using RNAzol (Takara) according to the manufacturer's instructions. Each RNA sample was eluted in 30 μL of RNase free water. The nucleic acid concentration and purity was determined using spectrophotometry. Total RNA (1 μg) was reverse transcribed to cDNA using oligo-dT primers and the PrimeScript RT-PCR Kit (Takara).

### Virus detection by RT-PCR

Two virus-specific RT-PCR assays were designed, based on primers located in the Helicase and RdRp domains (Figure S1). Amplifications were carried out in 20 μL total reaction volume using Ex-Taq Polymerase (Takara).The final PCR reaction contained 20 mM of Tris-HCl pH 8.4, 50 mM of KCl, 1.5 mM of MgCl_2_, 0.25 mM of dNTPs mix PCR grade, 0.4 μM of each primer, 2.5 U of Ex-Taq Polymerase and 2 μL of cDNA. The cycling protocol was: 94°C for 5 min, followed by 35 cycles of [94°C for 30 s; 48°C (Hel) or 52°C (RdRp) for 30 s; 72°C for 60 s] followed by a final extension of 72°C for 7 min. The assays were used to determine the presence of the virus in 12 individual samples each of *A. pernyi* larvae, pupae (chrysalis) and adults (moths), and 80 eggs; all from the Liao Ning province. The Helicase assay was also used to amplify PCR products for determination of virus variability by sequencing 9 isolates from the three provinces surveyed (Liao Ning, Ji Lin and He Nan).

## Results

### Genome analysis

The new virus genome is 10,163 nucleotides long, has a natural poly(A) tail at its 3′ end and contains a single large (9,108 nucleotide) open reading frame (ORF), flanked on the 5′ and 3′ by untranslated regions (UTR) of 883 nucleotides (5′UTR) and 172 nucleotides (3′UTR) in length, accounting for 10.3% of the genome. The genome is A/U rich (63.77%), which is consistent with that of other iflaviruses (e.g., KV 61.57%, DWV 61.26%, and VDV-1 61.41%) [11]–[13]. The presence of a poly(A) tail was confirmed by 3′RACE-PCRamplification.

The ORF is predicted to encode a 3036 amino acid polyprotein. No other ORF that could encode proteins larger than 70 amino acids were found, on either strand, confirming that the virus has a positive-strand RNA genome. The polyprotein contains a number of conserved domains:

Two Picornavirus-like capsid protein domains were identified between the amino acids 333–562 and 657–851 of the ApIV polyprotein, corresponding to VP3 and VP1, respectively, as well as a cricket paralysis virus (CrPV) capsid protein-like domain [14] between the amino acids1057 and 1291, corresponding to VP2 (Figure 3A). The boundaries of these domains correspond closely to the putative 3C-protease and autocatalytic cleavage sites (Figure 3A) that process the structural precursor protein into functional units during virus assembly.

An RNA helicase was identified between residues 1602 and 1753 (Figure 3A) including the highly conserved Hel-A motif (^1616^GxxExGKS^1623^) but with E^1619^ substituting the more common glycine (G) residue, which suggested to be responsible for nucleotide binding [15]. The other two helicase motifs, Hel-B (Qx5DD) and Hel-C (KGx4Sx5STN), are the same as the other Iflaviruses.

A conserved 3C cysteine protease domain was identified between amino acids 2428 and 2562 of the polyprotein (Figure 3A) with the conserved GxCG and GxHxxG motifs [16] matching the consensus sequence for the Picornavirales (Figure S2B).

This is followed by the eight conserved RdRp domains [17], [18], between amino acids 2700 and 2993 of the polyprotein, near the C- terminus (Figure 3A), including the acid motif V (^2795^SGx3Tx3N^2808^), the core motif VI (^2890^YGDD^2893^) for nucleotide binding, and motif VII (^2942^FLKR^2945^) for catalytic function [17] (Figure S2C).

The 5′UTR of ApIV is 883 nt long, and contains a number of stable secondary structures, clustered around 300 nt prior to the start of the ORF (Fig. 3B), including five hairpin structures (I–III; V–VI) and one Y-shaped structure (IV). They are similar to the IRES-related structures described for DWV/VDV-1 [5] in number, overall shape, distance relative to each other and distance from the ORF, and may well serve a similar function. Although the 5′ UTR of ApIV has only 32% nucleotide identity with the 5′UTR of VDV-1 and DWV, its closest relatives, there is considerable conservation of their predicted structural elements, supporting a functional role for these structures.

### Phylogenetic analysis

**Figure 4:**
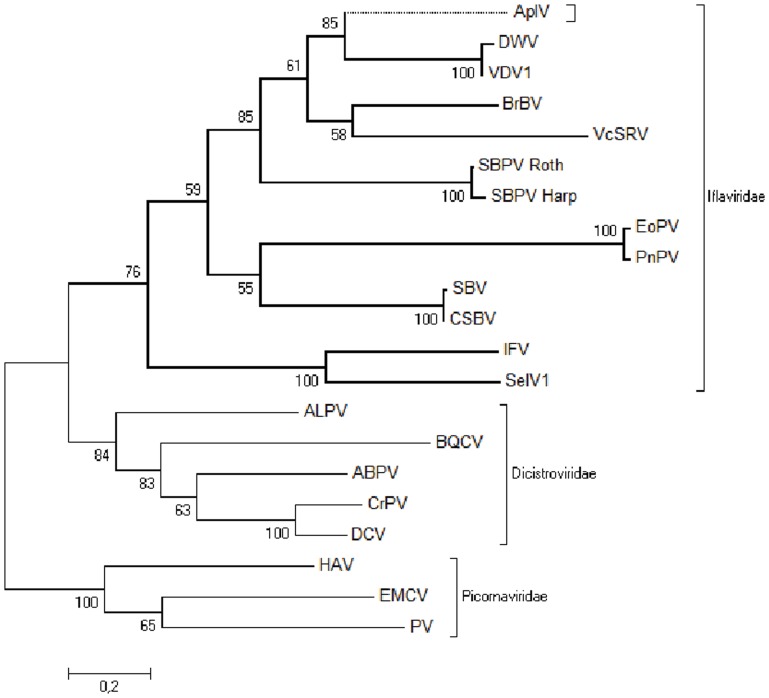
Phylogenetic analysis of ApIV with respect to viruses belonging to Iflaviridae as well as representative viruses from and the Dicistroviridae and the Picornaviridae (outgroup). The phylogeny is based on a concatenated 328 amino acid sequence combining the conserved domains of the helicase, protease and RdRp regions and was inferred by Maximum Likelihood using the JTT matrix-based model [39] as implemented by MEGA5 [40]. The tree with the highest log likelihood is shown. The percentage of trees in which the associated taxa clustered together is shown next to the branches. The full names and GenBank accession numbers of the viruses used are shown in Table S1.

The highly conserved motifs of the Helicase, 3C-protease and RdRp amino acid sequences from 19 viruses of the picorna-like superfamily were used for a phylogenetic analysis to evaluate the relationship of ApIV to other viruses. The RdRp has been used as a reliable protein to construct phylogenetic trees for classification of RNA viruses, as it tends to be highly conserved among RNA viruses [19], [20]. The Helicase and 3C-protease domains were included to widen the coverage of the phylogeny to other regions of the genome. Only the core motifs of the various domains were included, to ascertain positional homology throughout the alignment. The RdRp tree segregated the viruses into two groups according to their taxonomic classification, Iflaviridae and Dicistroviridae (Figure 4). ApIV is most closely related to the DWV/VDV-1 species complex, but distinct enough to be considered a new species within the Iflaviridae, which we propose to name *Antheraea pernyi* iflavirus (ApIV).

### Propagation, purification and infection of ApIV

**Figure 5:**
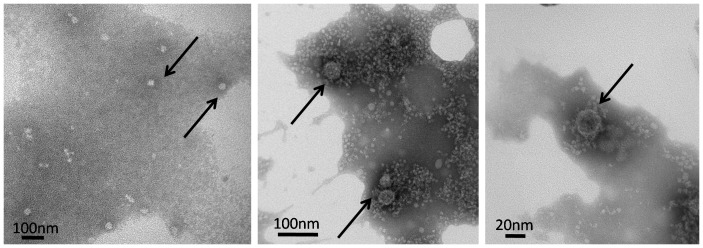
Electron micrograph of negatively stained virus particles purified by sucrose gradient centrifugation. Arrows indicate spherical virus particles of about 25–30 nm in diameter.

**Figure 6:**
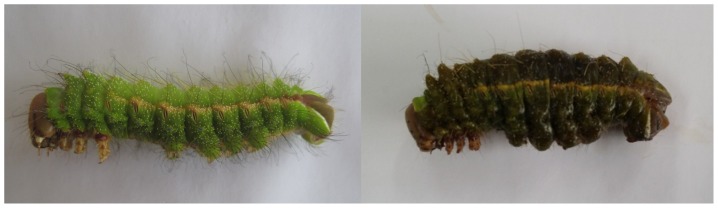
Phenotype of 5^th^ instar larvae injected with purified ApIV. Larvae at day 4 after injecting PBS (left) or ApIV diluted in PBS (right).

The internal tissues of AVD-symptomatic larvae were homogenized and injected into healthy pupae, to propagate any viruses present. The hemolymph of these infected pupae was extracted three days post-infection and subjected to discontinuous sucrose gradient centrifugation. This yielded a fraction containing Iflavirus-like particles (Fig. 5) and large amounts of ApIV RNA, as determined by RT-qPCR. Healthy *A. pernyi* larvae injected with this fraction reproduced typical AVD symptoms within 3 days after infection (sluggish movement, reduced ability to clasp branches, and darkened pygidia and head), after which their bodies turned black and they died (Fig. 6). Mock-injected larvae had normal phenotypes.

### Detection of ApIV in different life stages

Different life stages of healthy, asymptomatic *A. pernyi* from the Liao Ning province were assessed for the presence of ApIV through RT-PCR (Table 1). ApIV is most prevalent in larvae (3/12) followed by pupae (2/12), adults (1/12) and is least prevalent in eggs (0/80). This could suggest that the ApIV-infection is acquired during the larval stage.

**Table 1:** Presence of ApIV in asymptomatic *A. pernyi* of different developmental stages^a^.

Insect stage	ApIV positive
Egg	0/80^b^
Larva	3/12
Chrysalis	2/12
Moth	1/12

a all from the Liao Ning Province.

b eggs collected from 12 females.

### Prevalence and genetic variability of ApIV from different geographical regions

To determine the prevalence of ApIV throughout the natural range of *A. pernyi*, 40 healthy chrysalises collected from the Liao Ning and Ji Lin provinces in the north of China and He Nan province further south were assessed for the presence of ApIV. ApIV was detected in 22% of the chrysalises with similar prevalence in the three provinces studied.

A 420 bp section of the ApIV Helicase region and a 2129 bp section of the ApIV structural protein region was amplified and sequenced from 9 ApIV-positive chrysalises from the 3 different provinces studied: 4 from Ji Lin, 2 from Liao Ning and 3 from He Nan. Comparison of the sequences revealed low genetic variability of ApIV from the different geographic areas. There are only three variable nucleotide sites in the 420 bp helicase region, none of which affect the amino acid sequence (Figure S3) and only 4 variable nucleotide sites in the 2129 bp structural protein region, 2 of which lead to amino acid changes: a Leucine-Serine change at amino acid 367 and a Valine-Isoleucine change at amino acid 376 (Figure S4). In all, the ApIV isolates investigated in this study showed more than 99% nucleotide identity for these genomic regions, across all three provinces.

## Discussion

In this study, we report the discovery of a putative iflavirus from larvae affected by *A. pernyi* vomit disease (AVD), which we propose to call *Antheraea pernyi* iflavirus (ApIV). The virus is monocistronic, with a single-stranded RNA genome of at least 10,163 nucleotides, excluding the poly(A) tail and contains a single, large open reading frame encoding a 3,036 amino acid polyprotein containing domains for both structural and non-structural replication proteins. The similarity of the genome organization and amino acid sequence with the viruses of the genus *iflavirus* suggests that ApIV is a novel member of this genus.

The length of the 5′UTR length of ApIV (883 nt) is distinctly longer than that from other lepidopteran-infecting iflaviruses, such as *Ectropis oblique* picorna-like virus (473 nt; [21]), and *Perina nuda* virus (390 nt; [22]), but shorter than that of the DWV-species complex (1117–1156 nt; [23]–[25]) to which they are most closely related. These differences could be due to incomplete sequencing of the 5′ ends for some of the viruses, or be of functional significance.

The Iflavirus genome lacks a 5′ cap structure to operate the initiation of protein synthesis, like all other Picornaviridae. The iflavirus genome uses an internal ribosome entry site (IRES) for translation initiation [26]–[31]. Three iflaviruses were reported for having IRES activity in insect cells. They are EoPV [32], VDV-1 [5], and PnV [22]. It is therefore likely that the ApIV 5′UTR also contains IRES elements, but definitive proof of this awaits functional analysis of the 5′UTR regions.

The high degree of similarity between the ApIV strains isolated from three geographical regions at least 600 km apart indicate that the natural variability of ApIV is relatively is low, certainly compared to the honeybee Iflaviruses DWV/VDV-1 [23], [24], [33], [34]; SBPV [28] and SBV/CSBV [35], [36]. Millán-Leiva *et al.* [37] also found a high similarity between strains of SeIV (0.4% single nucleotide polymorphism; 39/10,347 bp), but these were isolated from the same laboratory and therefore raised the question of how large the variation is in nature. Our data show an SNP level of 0.7% (3/420 bp) and 0.2% (4/2129 bp) in geographically distantly separated isolates suggesting that the low variability could be an inherent property of the virus itself.

We were able to detect ApIV in AVD-affected larvae, and have shown that injection of purified ApIV leads to AVD symptoms, but the tissue distribution and the transmission routes of both the disease and the virus remain to be determined.

## Supporting Information

Figure S1 **Primers and primer locations.**
(DOCX)

Figure S2 **Amino acid sequence alignment of non-structural peptides.** (A) Helicase domain alignment. Helicase motifs conserved in Iflaviridae, (A–C) are indicated. (B) 3C-protease domain alignment. Conserved motifs (CxXG and GxHxxG) are indicated. (C) RdRp domain alignment. Eight conserved regions (I–VIII) are indicated. Black shading indicates 100% sequence identity and pink shading indicates 75% identity. The full virus names and the GenBank accession numbers for their nucleotide and amino acid sequences are given in Table S1.
(PPTX)

Figure S3 **Variation in the Hel region.** Multiple sequence alignment of the 420 bp Hel region (nucleotide position 5560–6080) of ApIV isolates from three different provinces in China, amplified by the primer pair Hel-F and Hel-R. The data are based on sequences from a total of 9 chrysalises (4 from Ji Lin, 2 from Liao Ning and 3 from HeNan).
(DOCX)

Figure S4 **Variation in the structural protein region.** Multiple sequence alignment of a 2127 bp region spanning VP3, VP4 and VP1 (nucleotide position 1784–3912) of ApIV isolates from three different provinces in China. The data are based on sequences from a total of 9 chrysalises (4 from Ji Lin, 2 from Liao Ning and 3 from HeNan). A, nucleotide alignment. B, amino acid alignment.
(PPTX)

Table S1 **Nucleotide sequence GenBank accession numbers and general classification of the picorna(-like) viruses discussed in this paper**
(DOCX)
