# Supplementary material for: Genetic Characterization of a Novel Iflavirus Associated with Vomiting Disease in the Chinese Oak Silkmoth Antheraea pernyi
Source: PLoS One. 2014 Mar 17;9(3):e92107. doi: 10.1371/journal.pone.0092107 (PMC3956879; doi:10.1371/journal.pone.0092107)
Supplement: Figure S1 — Primers and primer locations. (DOCX) [file pone.0092107.s001.docx]

Supplemental figure 1.

NS-R

RdRp-R

Hel-R

RdRp-F

NS-F

Hel-F

DP-F1 DP-F2

3’RACE

5’RACE

AAAn

RV-5 RV-4 RV-3 RV-2 RV-1

Schematic of relative positions of the primers used for cDNA synthesis from positive strand genomic RNA, and amplification of the ApIV genome. Arrows represent the primers used for amplifying the different domain of ApIV genome.

Primer sets used to clone, sequence, and amplify the ApIV genome.

| **Primer** | **Remarks** | **Sequence (5'-3')** |
| --- | --- | --- |
| DP-F1 | Forward primer for structural protein | CCTAA(C/T)ACTATTCC(A/T/C/G)TTTA |
| DP-F2 | Forward primer for structural protein | TATAA(C/T)CC(A/C)GAAAT(T/A)TT |
| RV-1 | Reverse primer for structural protein | CGCCTTGTACGGAATCCTCA |
| RV-2 | Reverse primer for structural protein | ATCTAACATCTGCAGACGGT |
| RV-3 | Reverse primer for structural protein | CGCCTTGTACGGAATCCTCA |
| RV-4 | Reverse primer for structural protein | TGCGAAGATAGTCCAAAAGC |
| RV-5 | Reverse primer for structural protein | TAGGACAATATGCGCAACAT |
| Hel-F | Forward primer for Hel domain | GTTTCCTGGTATTATTCA |
| Hel-R | Reverse primer for Hel domain | ACGCCATAATGCTATTTG |
| NS-F | Forward primer for Non-structural protein | AATGAATCCAACGTGTGG |
| NS-R | Reverse primer for Non-structural protein | GGACAATAGCACACGGCGAATCAA |
| RdRp-F | Forward primer for RdRp domain | ACTTATTGTGTGTGA |
| RdRp-R | Reverse primer for RdRp domain | ACATCGCTCTTATCT |
