# Supplementary figures and images for: Genetic Characterization of a Novel Iflavirus Associated with Vomiting Disease in the Chinese Oak Silkmoth Antheraea pernyi
Source: PLoS One. 2014 Mar 17;9(3):e92107. doi: 10.1371/journal.pone.0092107 (PMC3956879; doi:10.1371/journal.pone.0092107)

## Slide 1
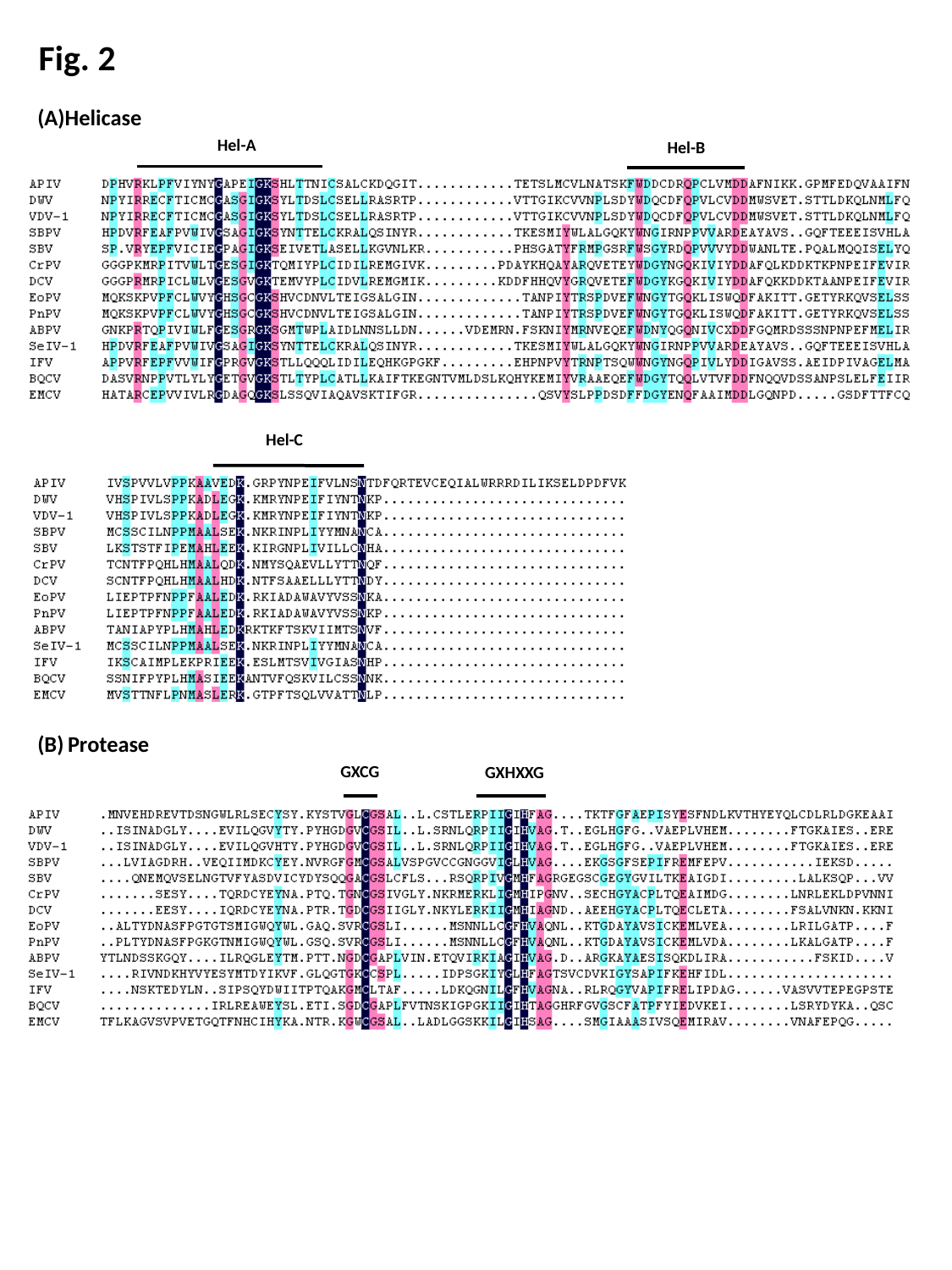

Fig. 2

## Slide 2
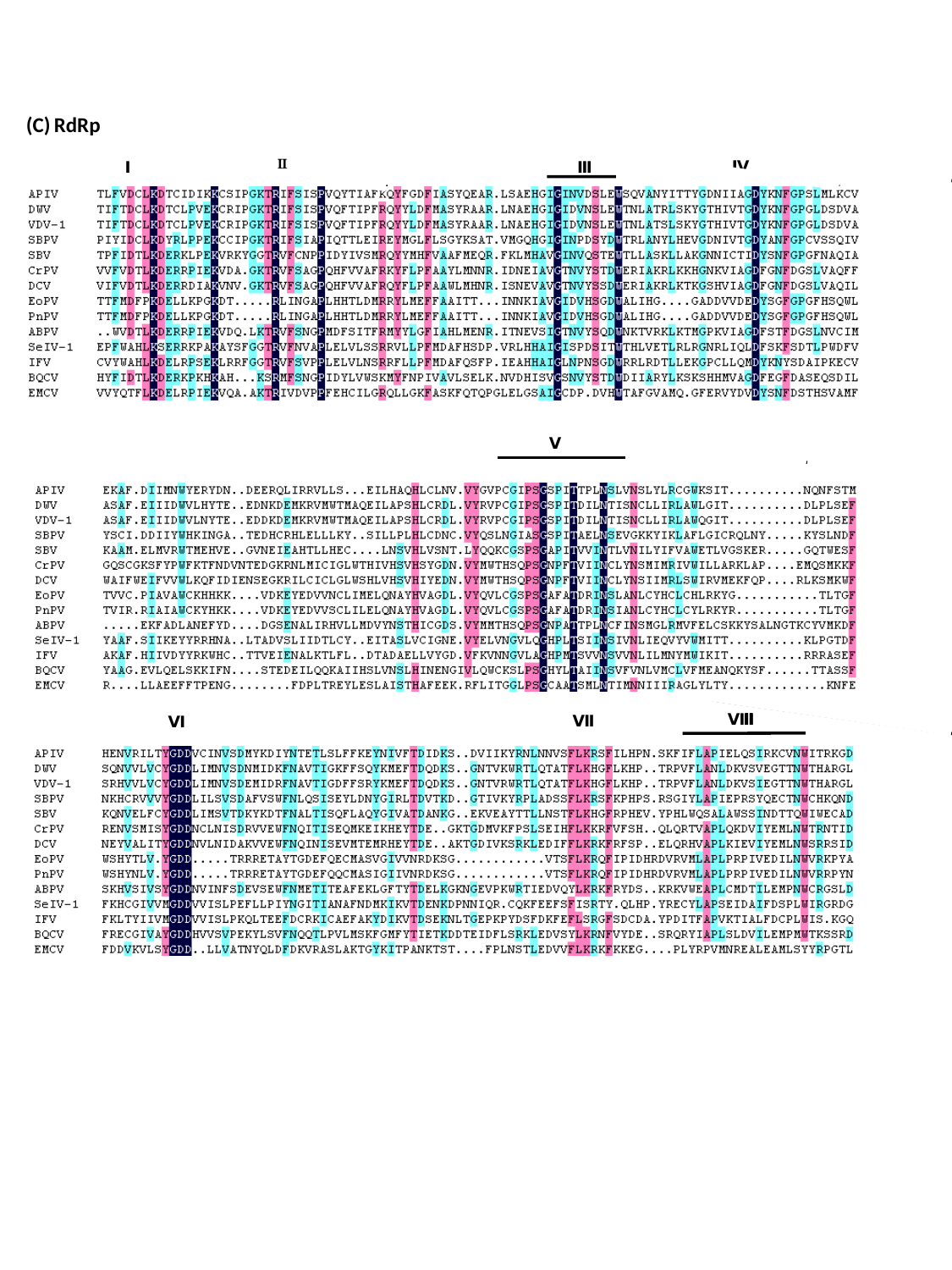

Supplement: Figure S2 — Amino acid sequence alignment of non-structural peptides. (A) Helicase domain alignment. Helicase motifs conserved in Iflaviridae, (A–C) are indicated. (B) 3C-protease domain alignment. Conserved motifs (CxXG and GxHxxG) are indicated. (C) RdRp domain alignment. Eight conserved regions (I–VIII) are indicated. Black shading indicates 100% sequence identity and pink shading indicates 75% identity. The full virus names and the GenBank accession numbers for their nucleotide and amino acid sequences are given in Table S1. (PPTX) [file pone.0092107.s002.pptx]

Fig. 4


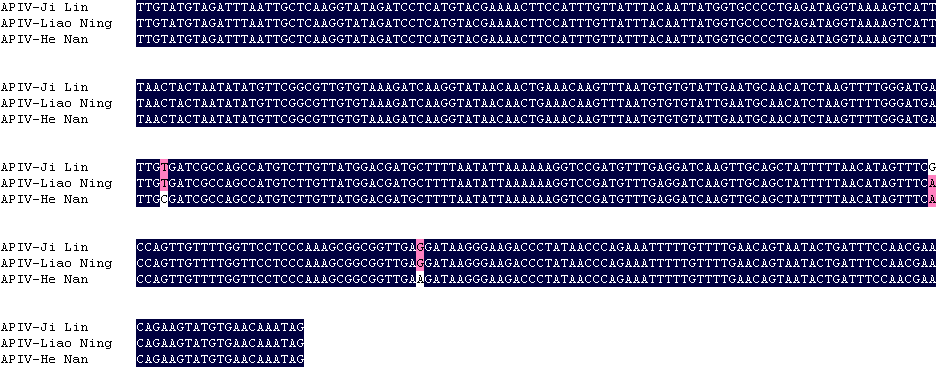

Supplement: Figure S3 — Variation in the Hel region. Multiple sequence alignment of the 420 bp Hel region (nucleotide position 5560–6080) of ApIV isolates from three different provinces in China, amplified by the primer pair Hel-F and Hel-R. The data are based on sequences from a total of 9 chrysalises (4 from Ji Lin, 2 from Liao Ning and 3 from HeNan). (DOCX) [file pone.0092107.s003.docx]

## Slide 1
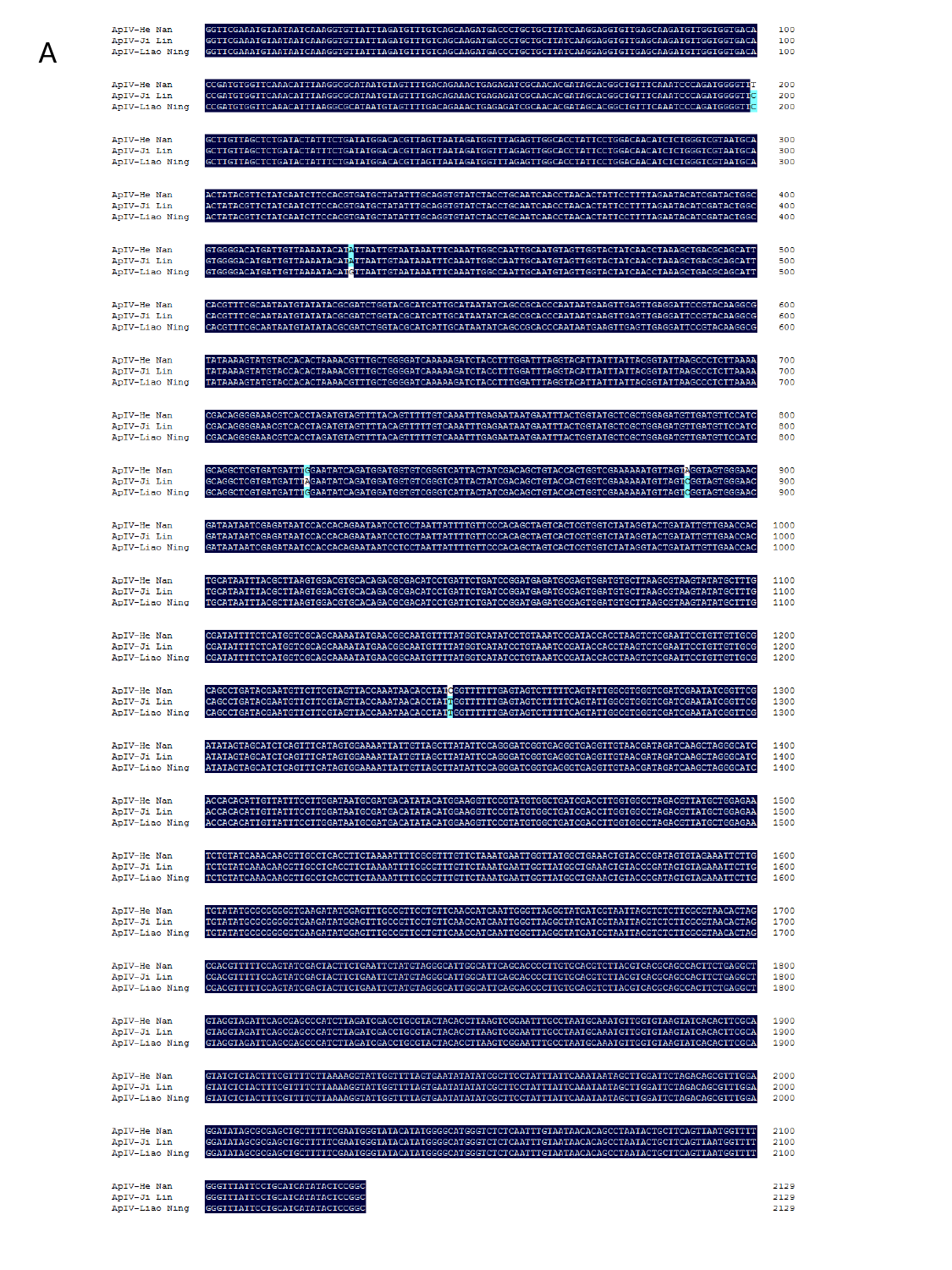

A

## Slide 2
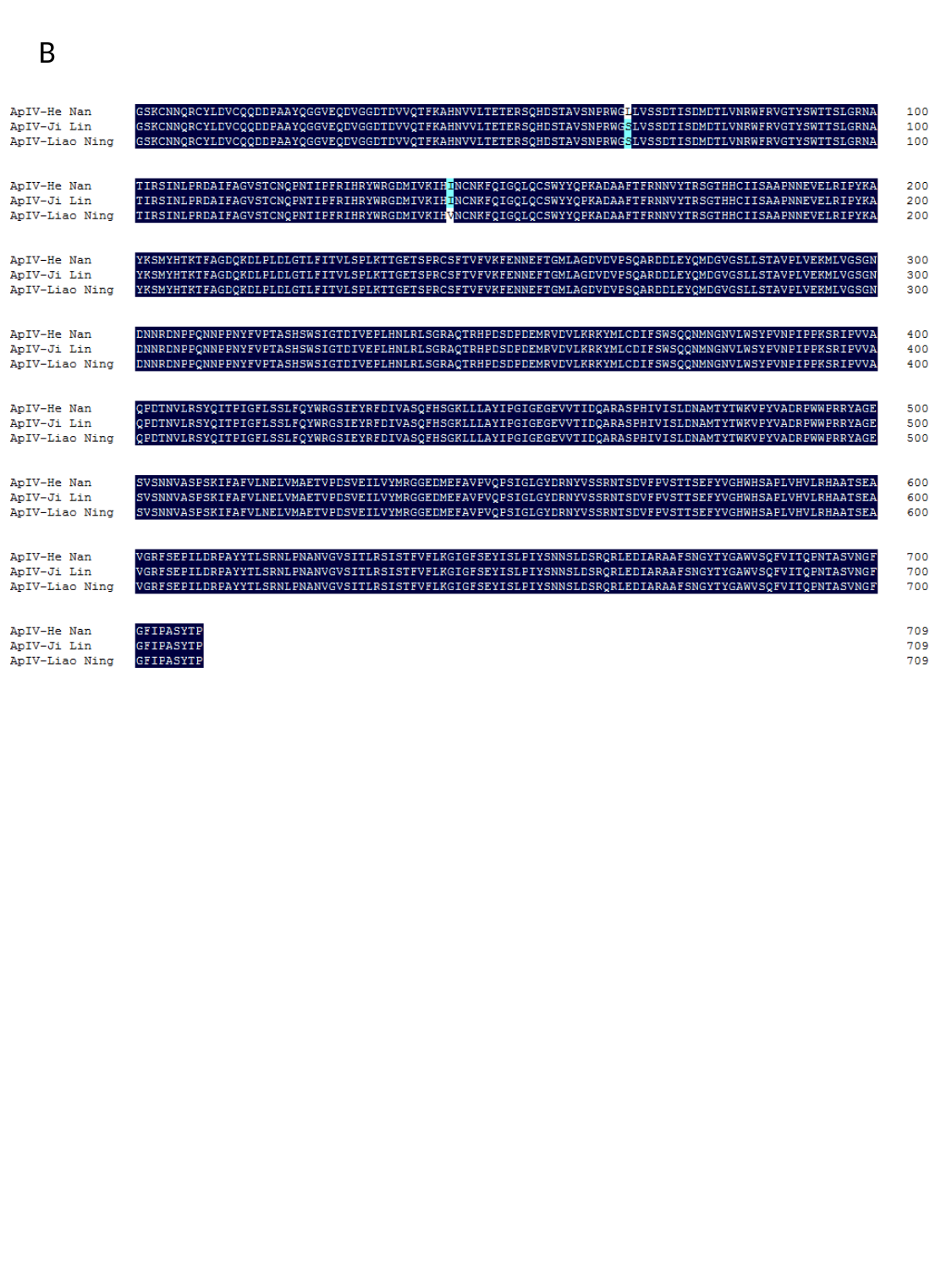

B

Supplement: Figure S4 — Variation in the structural protein region. Multiple sequence alignment of a 2127 bp region spanning VP3, VP4 and VP1 (nucleotide position 1784–3912) of ApIV isolates from three different provinces in China. The data are based on sequences from a total of 9 chrysalises (4 from Ji Lin, 2 from Liao Ning and 3 from HeNan). A, nucleotide alignment. B, amino acid alignment. (PPTX) [file pone.0092107.s004.pptx]
