# Supplementary material for: Genetic Characterization of a Novel Iflavirus Associated with Vomiting Disease in the Chinese Oak Silkmoth Antheraea pernyi
Source: PLoS One. 2014 Mar 17;9(3):e92107. doi: 10.1371/journal.pone.0092107 (PMC3956879; doi:10.1371/journal.pone.0092107)
Supplement: Table S1 — Nucleotide sequence GenBank accession numbers and general classification of the picorna(-like) viruses discussed in this paper (DOCX) [file pone.0092107.s005.docx]

Supplementary table 1. Nucleotide sequence GenBank accession numbers and general classification of the picorna(-like) viruses discussed in this paper

| **Virus** | | | | | **GenBank accession No.** | | |
| --- | --- | --- | --- | --- | --- | --- | --- |
| **Family** |  |  | ***Dicistroviridae*** |  |  |  |  |
| DCV |  |  | Drosophila C virus |  | AF014388 | | |
| CrPV |  |  | Cricket paralysis virus |  | AF218039 | | |
| BQCV |  |  | Black queen cell virus |  | AF183905 | | |
| ABPV |  |  | Acute bee paralysis virus |  | AF150629 | | |
| ALPV |  |  | Aphid lethal paralysis virus |  | AF536531 | | |
| **Family** |  |  | ***Iflaviridae*** |  |  |  |  |
| ApIV |  |  | Antheraea pernyi iflavirus |  | KF751885 | | |
| DWV |  |  | Deformed wing virus |  | AY292384 | | |
| IFV |  |  | Infectious flacherie virus |  | AB000906 | | |
| SBV |  |  | Sacbrood virus |  | AF092924 | | |
| BrBV |  |  | Brevicoryne brassicae picorna-like virus |  | EF517277 | | |
| PnPV |  |  | Perina nuda picorna-like virus |  | AF323747 | | |
| EoPV |  |  | Ectropis obliqua picorna-like virus |  | AY365064 | | |
| VDV-1 |  |  | Varroa destructor virus-1 |  | AY251269 | | |
| SeIV-1 |  |  | Spodoptera exigua iflavirus 1 |  | NC_016405.1 | | |
| SBPV |  |  | Slow bee paralysis virus |  | EU035616 | | |
| VcSRV |  |  | Venturia canescens small RNA virus |  | AY534885 | | |
| CSBV |  |  | Chinese sacbrood virus |  | AF469603 | | |
| **Family** |  |  | ***Picornaviridae*** |  |  |  |  |
| EMCV |  |  | Encephalomyocarditis virus |  | M81861 | | |
| HAV |  |  | Hepatitis A virus |  | K02990 | | |
| PV |  |  | Polio virus |  | CAA24465 | | |
